# Human eyes with dilated pupils induce pupillary contagion in infants

**DOI:** 10.1038/s41598-017-08223-3

**Published:** 2017-08-29

**Authors:** Christine Fawcett, Melda Arslan, Terje Falck-Ytter, Herbert Roeyers, Gustaf Gredebäck

**Affiliations:** 1 Uppsala University, Uppsala, Sweden; 2 Ghent University, Gent, Belgium; 3 Karolinska Institute, Solna, Sweden; 4 Center for Psychiatry Research, Stockholm County, Sweden

## Abstract

Being sensitive and responsive to others’ internal states is critical for social life. One reliable cue to what others might be feeling is pupil dilation because it is linked to increases in arousal. When adults view an individual with dilated pupils, their pupils dilate in response, suggesting not only sensitivity to pupil size, but a corresponding response as well. However, little is known about the origins or mechanism underlying this phenomenon of pupillary contagion. Here we show that 4- to 6-month-old infants show pupillary contagion when viewing photographs of eyes with varying pupil sizes: their pupils dilate in response to others’ large, but not small or medium pupils. The results suggest that pupillary contagion is likely driven by a transfer of arousal and that it is present very early in life in human infants, supporting the view that it could be an adaptation fundamental for social and emotional development.

## Introduction

To be a successful member of a social group, one must be sensitive and responsive to others’ internal states. One reliable cue for what others might be feeling is pupil size because pupils dilate with increases in both positive and negative arousal^1,2^. When adults view an individual with dilated pupils, their pupils tend to dilate in response^3,4^. Thus this phenomenon of pupillary contagion suggests the possibility that individuals are not only sensitive to internal arousal states via the pupil, but that they can respond with an arousal state of their own. However there has been very little examination of pupillary contagion’s development^5^ – a focus which can give insight into its origins and underlying mechanism. In the current study, we aim to shed light on these issues by examining whether infants as young as 4 to 6 months of age show pupillary contagion when viewing photographs of adults’ eyes with varying pupil sizes.

Pupillary contagion has been shown to be related to social and group processes. For example, pupillary contagion is more likely to occur when the observed individual is trusted^3^, is part of one’s in-group^3,4^, or expresses sadness^6^. Further, an observer’s level of empathy predicts how sensitive they are to pupil size when judging others’ sadness^7^. Because of these relations, pupillary contagion has been proposed to be evolutionarily adaptive for life in social groups, allowing coordination of internal states and shared understanding between group members^4^. One important piece of evidence supporting pupillary contagion’s evolutionary origins is that even chimpanzees show pupillary contagion within, but not across, species^4^. Additional critical support would come from demonstrating that pupillary contagion is present in very young infants – at around 4 months of age when their visual acuity is only just becoming sufficient to reliably perceive pupil size differences^8,9^. That is, if pupillary contagion was a learned response based on associations over time between observations of dilated pupils in others and experiences of arousing situations that lead to pupil dilation in the infant as well, it would require some time to build up observations of these co-occurrences. Thus, pupillary contagion in infants as young as 4 months of age would support a view that pupillary contagion emerges with little to no learning based on visual experience. This further suggests that there could be evolutionary pressure for sensitivity and responsivity to others’ pupil size, capacities which could be particularly important for infants who are beginning to adapt to the social world.

A further important issue to examine is what specific psychological or physiological mechanism can account for pupillary contagion. One proposed mechanism is automatic mimicry. Much like people non-consciously mimic the posture or movements of those around them^10^, it has been proposed that people could also mimic others’ pupil size^4^. An alternative proposal is that pupillary contagion is driven by a transfer of arousal in which viewing another’s dilated pupils leads to arousal in the observer that then leads to pupil dilation, signifying the shared state of arousal^5^. This second proposal would be more in line with research in other species showing that alerting group members of danger – for example via tail flagging in deer^11^, vocal alarm calls in chipmunks^12^, or chemical signals in fish^13^ – leads to changes in behavior that are not mimicry of the alarm, but rather a response to it, perhaps motivated by increased anxiety^14^.

**Figure 1:**
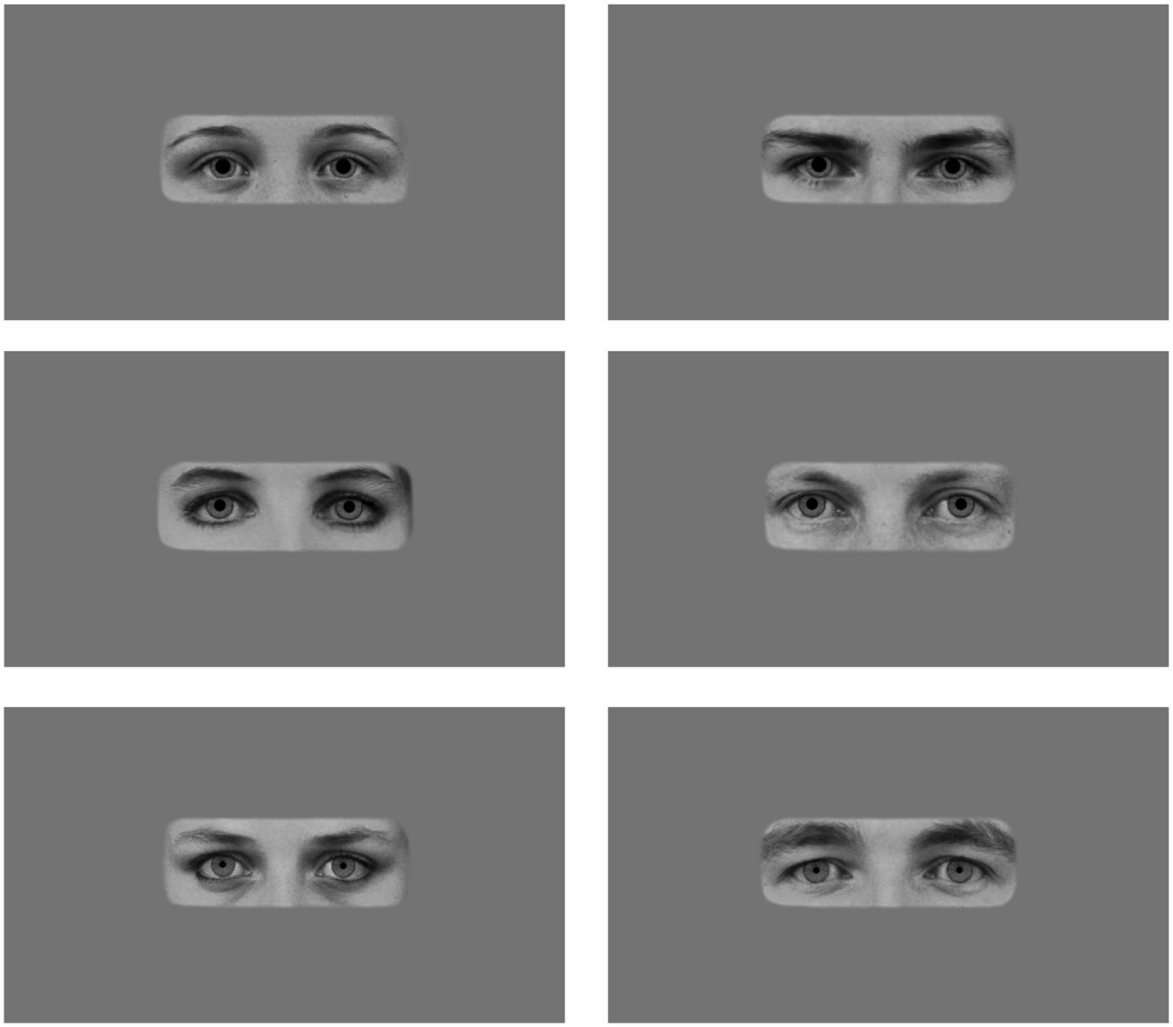
Stimuli depicting pupil sizes. Example images of female (left) and male (right) models with large, medium, and small (top to bottom) pupils as they were displayed on the screen. The complete set of 18 images included each of these six individuals with each of the three pupil sizes. Images were obtained from the Karolinska Directed Emotional Faces^26^ and modified in Adobe Photoshop (image IDs: BF19NES, BF13NES, BF01NES, AM14NES, AM10NES, and AM08NES; see Method for further details).

In the current study, infants at 4 and 6 months of age viewed a series of images of the eye region of male and female adults with small, medium, or large pupils (see Fig. 1). Infants’ own pupil dilation was measured using eye tracking in order to examine pupillary contagion’s ontogenetic origins, as well as to test two alternative views of the mechanism behind it. Specifically, if infants learn pupillary contagion through their visual experience, it either should not be present at all in such young infants, or should have significantly stronger effects in the 6-month-old group than in the 4-month-old group. Alternatively, finding pupillary contagion at this early age without such age differences would be more in line with the hypothesis that pupillary contagion is based on an evolved, adaptive mechanism that does not require learning. Regarding the psychological mechanism behind pupillary contagion, automatic mimicry would result in infants’ pupils being largest for the large-pupil images and smallest for the small-pupil images^3^. In contrast, an arousal mechanism would result in only the large-pupil images leading to larger pupil sizes in infants, with small- and medium-pupil images leading to similar pupil dilation^5^, given that arousal only leads to pupil dilation, not constriction^2,15^.

## Results

**Figure 2:**
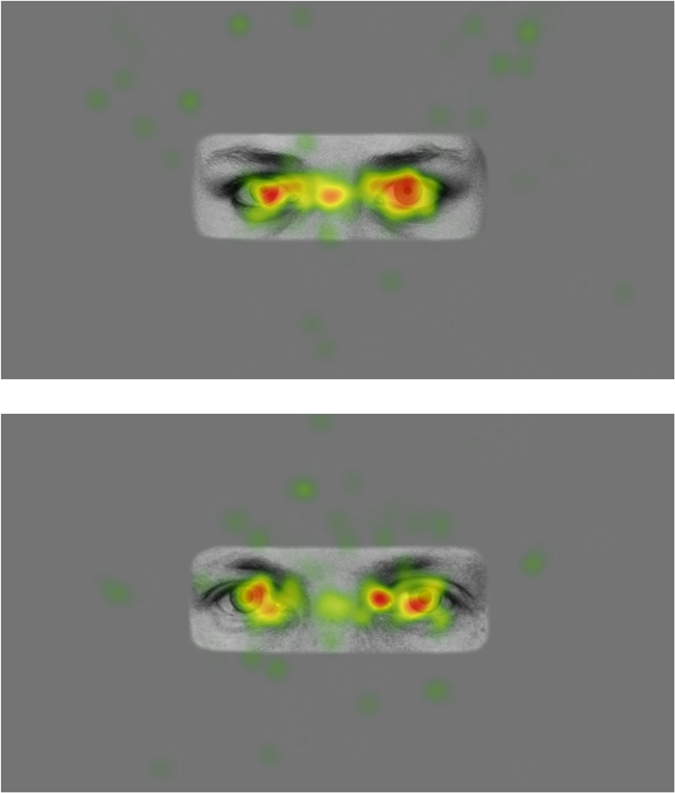
Example heat maps of infants’ gaze to stimuli.

Infants attended to the critical part of stimuli - the portion of the screen showing the image of the model - during 95.5% of their recorded gaze (see Fig. 2 for example heat maps). Stage 1 analyses of infants’ (*n* = 48) pupil size in reaction to viewing the images indicated that pupil size change in comparison to a baseline fixation screen was not significantly affected by image presentation order (*b* = −0.014, *SE* = 0.015, *t* = −0.932, *p* = 0.356), individual image brightness (*b* = 0.002, *SE* = 0.009, *t* = 0.192, *p* = 0.847), participant sex (*b* = 0.004, *SE* = 0.015, *t* = 0.273, *p* = 0.786), or the interactions between participant sex and model sex (*b* = −0.024, *SE* = 0.018, *t* = −1.299, *p* = 0.194), participant sex and age (*b* = 0.011, *SE* = 0.014, *t* = 0.749, *p* = 0.458), or participant sex and model pupil size (*Χ*^2^(2) = 1.162, *p* = 0.559). Thus image presentation order, image brightness, and participant sex were not included in the main analyses. There was, however, a significant effect of model sex on pupil size change (*b* = 0.045, *SE* = 0.009, *t* = 4.927, *p* < 0.001), so this variable was included in the main analyses.

Stage 2 (main) analyses for the pupillary contagion effect examined whether pupil size change from baseline to the 3-second analysis window was affected by the pupil size of the model (categorical variable: small, medium, or large), as well as the sex of the model (female or male), the age of the participant (4 or 6 months), and all interactions. These analyses revealed no significant interactions between predictors (size by sex by age: *Χ*^2^(2) = 4.530, *p* = 0.104; size by sex: *Χ*^2^(2) = 3.701, *p* = 0.157; size by age: *Χ*^2^(2) = 3.668, *p* = 0.160; sex by age: *b* = 0.009, *SE* = 0.009, *t* = 0.953, *p* = 0.341). However, when these non-significant interactions were removed from the model, and as predicted by the arousal view, infants’ own pupil size increases were affected by the model’s pupil size (*Χ*^2^(2) = 18.119, *p* < 0.001) such that they were greater when viewing an image with large pupils (*M*_*large*_ = 0.074 mm, *SD* = 0.173) compared to either medium (*M*_*medium*_ = 0.028 mm, *SD* = 0.153; *b* = −0.046, *SE* = 0.011, *t* = −4.158, *p* < 0.001) or small pupils (*M*_*small*_ = 0.042 mm, *SD* = 0.173; *b* = −0.032, *SE* = 0.011, *t* = −2.881, *p* = 0.004), and pupil size increases did not differ for medium compared to small pupils (*b* = −0.014, *SE* = 0.011, *t* = −1.278, *p* = 0.202; see Fig. 2). In addition, infants reacted to male models overall with greater pupil dilation than to female models (*M*_*male*_ = 0.070 mm; *M*_*female*_ = 0.025 mm; *b* = −0.044, *SE* = 0.009, *t* = −4.834, *p* < 0.001). Finally, there was a marginally significant difference between age groups such that 6-month-olds showed overall larger increases in pupil size from baseline than 4-month-olds did (*M*_*6 months*_ = 0.060 mm; *M*_*4 months*_ = 0.037 mm; *b* = −0.013, *SE* = 0.007, *t* = −1.772, *p* = 0.083). Given that this age effect did not interact with model pupil size, the results are in line with the adaptive view, rather than the learning view, of pupillary contagion. Examining the residuals for the main models lead to identification of some deviations from normality. To ensure that the results from our regression models were valid, we followed up our main analyses with non-parametric tests that are robust to such deviations. Results from these analyses lead to the same conclusions as the regression analyses, with the exception of the age effect which went from marginally significant to non-significant (see Supplementary Information).

**Figure 3:**
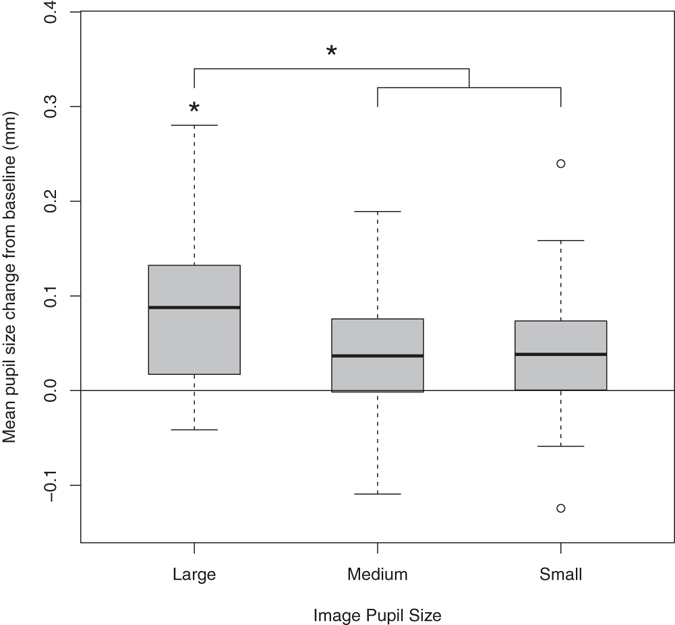
Box plot displaying average pupil size changes from baseline in response to viewing images varying in pupil size, age groups merged. Horizontal lines indicate medians, boxes indicate data within the 25^th^ to 75^th^ percentile, whiskers indicate data within the 5^th^ to 95^th^ percentile, and circles indicate outliers. Large-pupil images resulted in significantly more pupil dilation than either the medium- or small-pupil images and significant pupil dilation from baseline.

**Figure 4:**
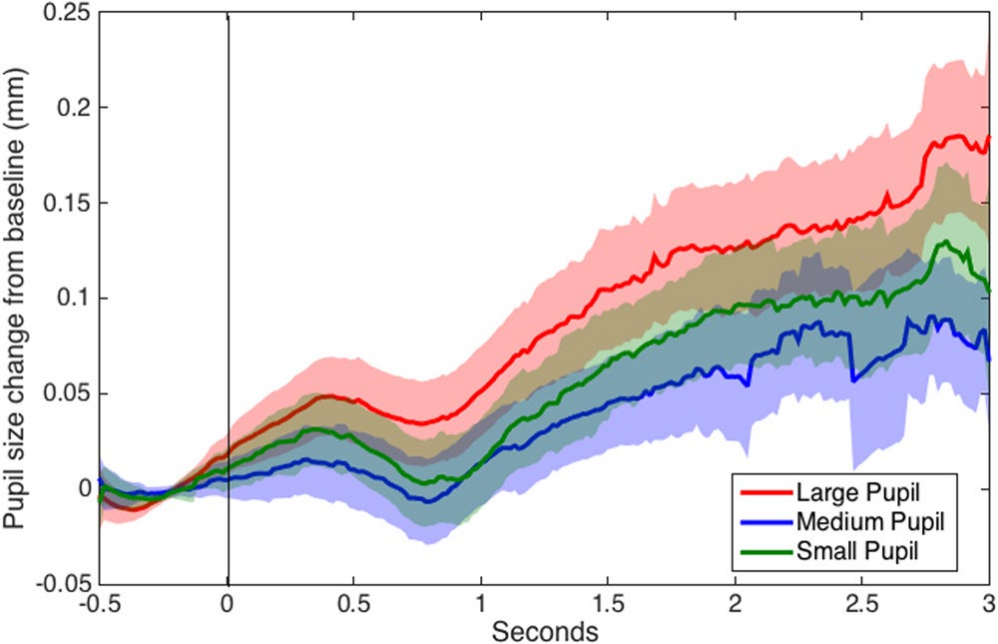
Recorded pupil size over time during viewing of images varying in pupil size (0 to 3 seconds) adjusted for baseline pupil size during fixation cross viewing (−0.5 to 0 seconds), age groups merged. Shaded regions indicate standard errors.

Stage 3 regression analyses examined whether significant pupil dilation occurred in response to each model pupil size compared to baseline. For each model pupil size, the intercept of a regression model with only random effects (for participant and trial) was examined. If the intercept of this model is significantly greater than zero, then the pupil difference from baseline is greater than zero. We found that there was significant pupil dilation in response to the images showing large (*b* = 0.076, *SE* = 0.013, *t* = 5.763, *p* < 0.001), but not medium (*b* = 0.027, *SE* = 0.016, *t* = 1.686, *p* = 0.129) or small (*b* = 0.041, *SE* = 0.022, *t* = 1.876, *p* = 0.110) pupils (see Figs 3 and 4). This provides further evidence that pupillary contagion occurs only in response to others’ dilated pupils. As with the main analyses, examination of residuals resulted in the identification of some deviations from normality and the regression was followed up with non-parametric tests. In this case, the non-parametric tests indicated significant dilation for each pupil image size. While this finding differs from the result from that of the parametric regression analysis, which found significant dilation only for large pupil images, it does not contradict the main finding that there was greater dilation for large pupils compared to the other sizes (see Supplementary Information for further analysis details).

A final analysis demonstrated that the pupil contagion effects could not be accounted for by difference in the individual images’ brightness levels by including the additional predictor of brightness in the main analysis (see Supplementary Results for further details).

## Discussion

Given that pupillary contagion suggests the ability for individuals to perceive and respond to the emotional states of others and to coordinate within social groups, it would be particularly important for infants’ development. Infants seem sensitive to others’ distress^16^ and can recognize facial expressions of emotion^17^ from an early age, however almost no studies have examined pupillary contagion in infancy. One previous study using schematic stimuli showed that 6- and 9-month-old infants’ pupil dilation was greater when viewing pairs of circles with large black centers than smaller black centers^5^. No comparable effect was found in a control condition with pairs of squares, leading the authors to suggest that this was the first evidence of pupillary contagion in infancy. While this initial study was important to rule out the effects of light as the cause of a pupillary contagion effect, it remained unclear whether infants truly viewed the schematic pairs of circles as eyes. This made further inquiries into this effect imperative to establish the basic pupillary contagion phenomenon in infants, an aim fulfilled by the current study.

The current results additionally help to elucidate the mechanism underlying pupillary contagion. While one possibility is that humans naturally and automatically mimic others, including their pupil size^4^, the current pattern of results supports an alternative proposal that pupillary contagion primarily stems from an automatic spreading of arousal across individuals^5^. Specifically, our results show pupillary contagion only to large pupils – an indicator of arousal – with no difference in reactions to small and medium pupils. This is the first study to investigate pupillary contagion to smaller and larger pupils in infants and even in research with adults, the question has been addressed only in a few studies and with mixed results. For example, Harrison and colleagues^7^ showed pupillary contagion only for larger and not for smaller pupils in adults, while Kret and colleagues^3^ seemed to show contagion to pupil constriction as well. However, it is important to note that these findings in adults could have been influenced by other factors in each experiment, such as emotional expressions^7^ or whether the observed individual was a member of another race^3^, making it more difficult to use them to draw clear conclusions about underlying mechanisms. In contrast, our study included only sex of model as an additional factor, and it was shown not to interact with the pupillary contagion effect. Thus our findings with infants are able to support arousal as the basic mechanism of pupillary contagion.

The early development of pupillary contagion provides evidence not only for its ontogeny, but also for its evolutionary origins. While a previous study showed that chimpanzees show pupillary contagion when viewing other members of their own species^4^, demonstrating that pupillary contagion is present in infants from just a few months of age also contributes support to the proposal that it is an adaptive ability rooted in evolutionary history.

One could argue that infants have the opportunity to gain significant amounts of social experience observing others’ pupil dilation even in just four months, opening up the possibility that general associative learning mechanisms could result in pupillary contagion, for example by observing dilated pupils in others during arousing situations. However, given how poor infants’ visual acuity is at birth, it is likely that they cannot perceive variation in others’ pupil size clearly until they are at least 3 to 4 months of age^8^. Exact estimates for this ability are difficult, given that others’ pupil sizes would be viewed in various lighting conditions and at various distances, but visual acuity norms for infants show that only by 3 to 4 months, can they perceive contrasting grids at a size comparable to the pupil size differences used in our study^9^. This factor would limit their potential to learn about pupil size and the situations that result in it, as well as limiting our ability to test whether pupillary contagion is present at earlier ages than those in the current study.

Together, the findings on pupillary contagion’s arousal mechanism and potential evolutionary history suggest the possibility that pupil dilation serves as a cue to synchronize arousal across group members for humans, similarly to how other species use visual^11^, vocal^12^, or chemical^13^ signaling to alert group members of danger and coordinate behavioral responses, possibly via synchronization of anxiety^14^. In this way, our findings help to inform our understanding of the commonalities across species in contagion of behavior and internal states.

One additional finding in our study was that infants responded to males’ eyes with greater pupil dilation than they did to females’ eyes overall. This result is not particularly surprising given that infants tend to spend more time with female caregivers overall^18,19^ and process female faces more readily during infancy^20,21^, especially when their caregiving is primarily from their mother^22^. Thus, their greater pupil dilation to males overall likely indicates increased attention and arousal to more novel stimuli.

A limitation of our study is that we do not have an additional measure of arousal to examine whether it is correlated with pupil dilation in the current paradigm. Such relations would provide additional support for the arousal view of pupillary contagion. However there is robust evidence that arousal leads to pupil dilation in both adults and infants, Specifically, adults’ pupils dilate when viewing emotionally arousing images^2^ or hearing emotionally arousing sounds^23^ and these dilations are related to other measures of arousal, such as skin conductance^2^ and self-ratings^23^. Further, 6- and 12-month-old infants’ pupils dilate when observing another infant’s expression of positive or negative emotion or when observing non-rational actions^24,25^. Thus, we believe that it is highly likely that arousal is involved in infants’ pupil dilation in this paradigm as well. Still, this is an important question for future research.

The current finding that infants as young as 4 to 6 months of age respond to others’ pupil dilation – a subtle cue of arousal – with pupil dilation of their own provides evidence for the view that pupillary contagion is rooted in a spontaneous transfer of arousal with potential evolutionary origins and adaptive outcomes. Specifically, this arousal transfer could further play a critical role in the development of social skills, such as empathy and group coordination.

## Method

### Participants

Twenty-four 4-month-olds (*M* = 4 months, 4 days, SD = 7 days; 11 female) and 24 6-month-olds (*M* = 6 months 5 days, *SD* = 7 days; 14 female) were included in the study. An additional 14 infants participated but were not included in the final analyses due to fussiness (*n* = 1 4-month-old), inability to calibrate gaze (*n* = 3 4-month-olds), or insufficient data (i.e., at least 4 trials with 50% or more pupil data collected per pupil size condition were required for inclusion; *n* = 2 4-month-olds and *n* = 8 6-month-olds; there was no inclusion criterion for number of trials with male and female models as this was not a main research question). Sample size was predetermined by estimation of likely effect sizes based on pilot data. Participants were recruited from a list of families that had expressed interest in participating in research with their child. The procedure was approved by the local ethical board (Regionala Etikprövningsnämnden) and caregivers provided written informed consent before beginning the experiment. All methods were performed in accordance with the relevant guidelines and regulations. Families received a gift voucher worth approximately 10 euros for their participation.

### Materials and Procedure

Infants sat on their caregiver’s lap approximately 50 cm from the screen of a Tobii T120 remote eye tracker, which was used to record their eye movements. The Tobii T120 has a freedom of head movement within an area of 40 × 20 × 27 cm. Gaze was recorded at 60 Hz. Before beginning the experiment, a 5-point calibration was used.

Images of three male and three female individuals with neutral facial expressions were selected from the Karolinska Directed Emotional Faces^26^ and modified in Adobe Photoshop (see Fig. 1; image IDs: BF19NES, BF13NES, BF01NES, AM14NES, AM10NES, and AM08NES). Faces were cropped to show only the eye region (from eyebrows to upper nose), made black and white. Next, the natural iris and pupil from each individual were replaced with an identical iris across all images and either a small, medium, or large pupil, following on the sizes used by Kret *et al*.^3^ in which the large pupil diameter was 40% larger and the small pupil diameter was 40% smaller than the diameter of the medium pupil. This resulted in pupils that were approximately 0.5, 0.8, and 1.1 visual degrees in diameter for the infant, differences which should be perceptible for both age groups^9^. Finally, the images were placed on a gray background (R, G, and B = 116) with a blurred edge to decrease distraction due to a sharp contrast at the edge of the photo. The gray background was selected to match the average brightness of the photos, as determined using the histogram tool in GIMP (GNU Image Manipulation Program, v. 2.8.18). Image brightness did not differ across pupil sizes (*M*_large_ = 116.03, *SD*_large_ = 0.59; *M*_medium_ = 116.12, *SD*_medium_ = 0.61; *M*_small_ = 116.15, *SD*_small_ = 0.58) or model sex (*M*_female_ = 116.20, *SD*_female_ = 0.36; *M*_male_ = 116.00, *SD*_male_ = 0.71). Images were displayed at near life-size on the screen, with the eye image portion 8 cm high by 23 cm wide.

The resulting 18 images (6 individuals with 3 pupil sizes each) were shown twice for 3 seconds each time, resulting in 36 trials. Each trial was preceded by a 1-second fixation screen with a small black cross shown on the same gray background as the test images, in order to minimize the pupillary light reflex upon seeing a new image^5,27^. To facilitate infants’ attention to the screen, every fourth trial was preceded by a short (2–3 second) animated video and every twelfth trial was preceded by a longer (7–10 second) animated video. Images were presented in one of two orders, each with the requirements that two images with the same pupil size were not shown consecutively and that the same individual was not shown consecutively. Total presentation time was approximately 3.5 minutes.

### Data reduction and analyses

Exported data files from the Tobii eye tracker were processed using Timestudio^28^, a MATLAB-based open access analysis tool for time series data (MATLAB version 8.5.0, Timestudio version 3.15; timestudioproject.com; the exact analyses used can be downloaded from within the Timestudio program using uwid: ts-fbf-b8d). Gaps in the data of up to 10 samples were interpolated linearly (larger gaps were considered missing data) and the data was smoothed using a moving average over 5 samples. Next, the values for each trial were adjusted using a baseline from the 0.5 seconds before the trial began. That is, the last 0.5 seconds of the fixation cross screen became the baseline for the entire 3-second viewing of the eye image. This baseline helps to accommodate for natural variation in pupil size. Finally, individual trials that were missing more than 50% of data in the combined baseline and trial due to inattention or technical problems were excluded (4 months: *n* = 253 trials, 29.3%; 6 months: *n* = 227 trials, 26.3%). In addition, trials with a score more than 3 standard deviations from the mean were excluded (4 months: *n* = 2 trials, 0.3% of remaining trials; 6 months: *n* = 12 trials, 1.9% of remaining trials).

In order to be able to assess infants’ attention to the photograph of the models’ eyes, we also created an area of interest (AOI) around the eye image portion of the screen and calculated the percentage of gaze in this AOI compared to gaze to the screen overall.

Statistical analyses were performed in R^29^ (version 3.1.1) using the packages lmerTest (version 2.0) and PMCMR (version 4.1). Data were primarily analyzed using mixed-effect regression models. Common across all models was that pupil size change from baseline in individual trials was the dependent variable and that there were random intercepts for participant and trial. Random effects are beneficial for taking into account variability between individual participants and across trials, thus giving analyses greater power^30^. When a model included a fixed effect with more than two categorical levels (i.e. model pupil size large, medium, or small), overall significance of the effect was first tested with a model comparison Chi-square test between regression models with and without the fixed effect. If the model comparison was significant, the effects between levels of the variable were reported. For the Stage 2 and 3 analyses, if examination of residuals using quantile-quantile plots indicated the possibility of non-normal distribution, follow-up non-parametric tests (either Kruskal-Wallis or Friedman tests, depending on the variables to be included) were performed to ensure robustness of results.

### Data availability

The datasets generated and analysed during the current study, as well as statistical analysis code, are available from the corresponding author on reasonable request.

## Electronic supplementary material

Supplementary Results

## References

[1] Laeng B, Sirois S, Gredeback G (2012). Pupillometry: A Window to the Preconscious?. Perspect. Psychol. Sci..

[2] Bradley, M. M., Miccoli, L., Escrig, M. A. & Lang, P. J. The pupil as a measure of emotional arousal and autonomic activation. *Psychophysiology* **45**, 602–7 (2008). 10.1111/j.1469-8986.2008.00654.x PMC3612940 18282202

[3] Kret ME, Fischer AH, De Dreu CKW (2015). Pupil Mimicry Correlates With Trust in In-Group Partners With Dilating Pupils. Psychol. Sci..

[4] Kret ME, Tomonaga M, Matsuzawa T (2014). Chimpanzees and humans mimic pupil-size of conspecifics. PLoS One.

[5] Fawcett C, Wesevich V, Gredebäck G (2016). Pupillary Contagion in Infancy: Evidence for Spontaneous Transfer of Arousal. Psychol. Sci..

[6] Harrison NA, Wilson CE, Critchley HD (2007). Processing of observed pupil size modulates perception of sadness and predicts empathy. Emotion.

[7] Harrison NA, Singer T, Rotshtein P, Dolan RJ, Critchley HD (2006). Pupillary contagion: central mechanisms engaged in sadness processing. Soc. Cogn. Affect. Neurosci..

[8] Atkinson J, Braddick O, Moar K (1977). Development of contrast sensitivity over the first 3 months of life in the human infant. Vision Res..

[9] Salomao SR, Ventura DF (1995). Large sample population age norms for visual acuities obtained with Vistech-Teller Acuity Cards. Investig. Ophthalmol. Vis. Sci..

[10] Chartrand TL, Lakin JL (2013). The Antecedents and Consequences of Human Behavioral Mimicry. Annu. Rev. Psychol.

[11] Hirth DH, McCullough DR (1977). Evolution of Alarm Signals in Ungulates with Special Reference to White-Tailed Deer. Am. Nat..

[12] Weary DM, Kramer DL (1995). Response of eastern chipmunk to conspecific alarm calls. Anim. Behav..

[13] Smith RJF (1992). Alarm signals in fishes. Rev. Fish Biol. Fish..

[14] Izhar R, Eilam D (2010). Together they stand: A life-threatening event reduces individual behavioral variability in groups of voles. Behav. Brain Res..

[15] Gilzenrat MS, Nieuwenhuis S, Jepma M, Cohen JD (2010). Pupil diameter tracks changes in control state predicted by the adaptive gain theory of locus coeruleus function. Cogn. Affect. Behav. Neurosci..

[16] Dondi M, Simion F, Caltran G (1999). Can newborns discriminate between their own cry and the cry of another newborn infant?. Dev. Psychol..

[17] Grossmann T (2010). The development of emotion perception in face and voice during infancy. Restor. Neurol. Neurosci..

[18] Ramsey JL, Langlois JH, Marti NC (2005). Infant categorization of faces: Ladies first. Dev. Rev..

[19] Ramsey-Rennels JL, Langlois JH (2006). Infants’ differential processing of female and male faces. Curr. Dir. Psychol. Sci..

[20] Quinn PC (2010). Infant preference for individual women’s faces extends to girl prototype faces. Infant Behav. Dev..

[21] Quinn PC, Yahr J, Kuhn A, Slater AM, Pascalis O (2002). Representation of the gender of human faces by infants: A preference for female. Perception.

[22] Gredebäck G, Eriksson M, Schmitow C, Laeng B, Stenberg G (2012). Individual differences in face processing: Infants’ scanning patterns and pupil dilations are influenced by the distribution of parental leave. Infancy.

[23] Partala T, Surakka V (2003). Pupil size variation as an indication of affective processing. Int. J. Hum. Comput. Stud..

[24] Geangu E, Hauf P, Bhardwaj R, Bentz W (2011). Infant pupil diameter changes in response to others’ positive and negative emotions. PLoS One.

[25] Gredebäck G, Melinder A (2010). Infants’ understanding of everyday social interactions: A dual process account. Cognition.

[26] Lundqvist, D., Flykt, A. & Öhman, A. *The Karolinska Directed Emotional Faces - KDEF*. (CD ROM from Department of Clinical Neuroscience, Psychology section, Karolinska Institutet, 1998).

[27] Nyström P, Gredebäck G, Bölte S, Falck-Ytter T (2015). Hypersensitive pupillary light reflex in infants at risk for autism. Mol. Autism.

[28] Nyström P, Falck-Ytter T, Gredebäck G (2016). The TimeStudio Project: An open source scientific workflow system for the behavioral and brain sciences. Behav. Res. Methods.

[29] R Development Core Team. R: A language and environment for statistical computing. *Found. Stat. Comput. Vienna, Austria* (2005).

[30] Baayen RH, Davidson DJ, Bates DM (2008). Mixed-effects modeling with crossed random effects for subjects and items. J. Mem. Lang..

