# Author Correction: Human eyes with dilated pupils induce pupillary contagion in infants

**DOI:** 10.1038/s41598-018-22184-1

**Published:** 2018-03-02

**Authors:** Christine Fawcett, Melda Arslan, Terje Falck-Ytter, Herbert Roeyers, Gustaf Gredebäck

**Affiliations:** 1 Uppsala University, Uppsala, Sweden; 2 Ghent University, Gent, Belgium; 3 Karolinska Institute, Solna, Sweden; 4 Center for Psychiatry Research, Stockholm County, Sweden

Correction to: *Scientific Reports* 10.1038/s41598-017-08223-3, published online 29 August 2017

The Acknowledgements section in this Article is incomplete.

“This research was supported by the European Research Council (ERC StG CACTUS 312292) and the Swedish Research Council (2014-1156).”

should read:

“This research was supported by the European Research Council (ERC StG CACTUS 312292 and MSCA ITN Brainview 642996) and the Swedish Research Council (2014-1156).”

