# Supplementary material for: Human eyes with dilated pupils induce pupillary contagion in infants
Source: Sci Rep. 2017 Aug 29;7:9601. doi: 10.1038/s41598-017-08223-3 (PMC5574996; doi:10.1038/s41598-017-08223-3)
Supplement: Supplementary file 1 — Supplementary Results [file 41598_2017_8223_MOESM1_ESM.pdf]

Human eyes with dilated pupils induce pupillary contagion in infants

Christine Fawcett[1], Melda Arslan[2], Terje Falck-Ytter[1,3,4], Herbert Roeyers[2] & Gustaf Gredebäck[1]

[1] Uppsala University

[2] Ghent University

[3] Karolinska Institute

[4]Center for Psychiatry Research, Stockholm County Council

Supplementary Results

Quantile-quantile plots of the residuals from the final model of the Stage 2 (main) analysis examining the effects of model pupil size, model sex, and participant age indicated some deviations from normality, as did the plots for the Stage 3 analyses examining whether pupil size changes differed from baseline (see Fig. S1), thus these regression models were followed-up by non-parametric tests robust to such violations.

Regarding the main analyses, for the within-subjects effect of model pupil size, a Friedman test on the average pupil size change values for each infant in response to each model pupil size was performed and size was found to be significant (*Friedman* $X^2(1) = 12.875$, $p = .002$). This was followed up by a posthoc Nemenyi multiple comparison test which replicated the main finding that infants reacted with greater pupil dilation to the large, compared to either the medium (*Nemenyi* = 4.763, $p = .002$) or small (*Nemenyi* = 3.897, $p = .016$) pupils, while there was no difference for medium compared to small pupil images (*Nemenyi* = 0.866, $p = .813$). For the within-subjects effect of model sex, a Friedman test was also used on the average pupil size changes for each infant in response to male and female models. Again, the significant effect was replicated (*Friedman* $X^2(1) = 30.083$, $p < .001$). For the age effect, a Kruskal-Wallis test was performed on the overall average pupil size change, comparing the two between-subjects age groups. Here, the marginal effect from the regression was not replicated and the effect was instead non-significant (*Kruskal-Wallis* $X^2(1) = 1.966$, $p = .161$).

We followed up our Stage 3 analyses with non-parametric one-sample tests. Specifically, Wilcoxon Signed Rank tests indicated that pupil size change from

baseline was significantly greater than zero for each model pupil size (Large: V = 1121, $p < .001$; Medium: V = 904, $p < .001$; Small: V = 997, $p < .001$).

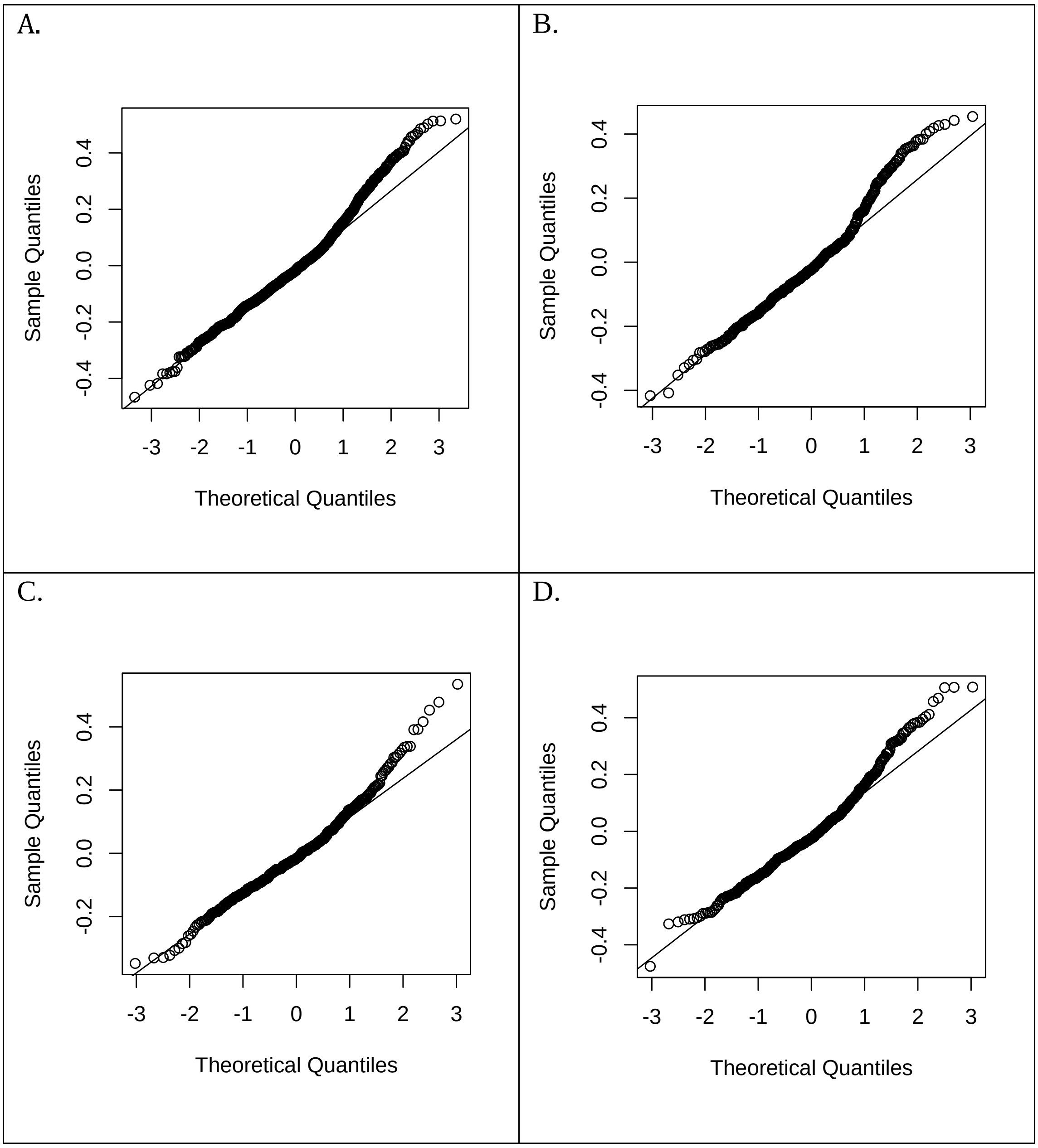

Figure S1. Quantile-quantile plots for the residuals of the main regression analysis (A), and the secondary regression analyses for large (B), medium (C), and small (D) model pupil sizes. In each case, there is some deviation from normality.

Analyses to examine brightness effects were also conducted to rule out the effect of light in creating the pupil contagion effect. The main regression analyses were repeated with individual images' brightness levels as an additional predictor. These regression models showed that brightness did not significantly affect pupil size over our analysis window ($X^2(1) = 2.570$, $p = .109$) and that the other reported effects remained significant. Specifically, model's pupil size was significant ($X^2(2) = 19.084$, $p < .001$) in that pupils were larger when viewing an image with large pupils compared to either medium ($b = -0.048$, $SE = .013$, $t = -4.302$, $p < .001$) or small pupils ($b = -0.032$, $SE = .011$, $t = -2.900$, $p = .004$), and pupil size increases did not differ for medium compared to small pupils ($b = -0.016$, $SE = .011$, $t = -1.284$, $p = .200$). In addition, infants reacted to male models overall with greater pupil dilation than to female models ($b = -0.047$, $SE = .009$, $t = -5.073$, $p < .001$) and there was a marginally significant difference between age groups such that 6-month-olds showed overall larger increases in pupil size from baseline than 4-month-olds did ($b = -0.013$, $SE = .007$, $t = -1.764$, $p = .084$).
